# Variations in the transcriptome of Alzheimer's disease reveal molecular networks involved in cardiovascular diseases

**DOI:** 10.1186/gb-2008-9-10-r148

**Published:** 2008-10-08

**Authors:** Monika Ray, Jianhua Ruan, Weixiong Zhang

**Affiliations:** 1 Washington University School of Engineering, Department of Computer Science and Engineering, 1 Brookings Drive, Saint Louis, Missouri 63130, USA; 2 University of Texas at San Antonio, Department of Computer Science, One UTSA Circle, San Antonio, Texas 78249, USA; 3 Washington University School of Medicine, Department of Genetics, 660 S. Euclid Ave, Saint Louis, Missouri 63110, USA

## Abstract

**Background:**

Because of its polygenic nature, Alzheimer's disease is believed to be caused not by defects in single genes, but rather by variations in a large number of genes and their complex interactions. A systems biology approach, such as the generation of a network of co-expressed genes and the identification of functional modules and *cis*-regulatory elements, to extract insights and knowledge from microarray data will lead to a better understanding of complex diseases such as Alzheimer's disease. In this study, we perform a series of analyses using co-expression networks, *cis*-regulatory elements, and functions of co-expressed gene modules to analyze single-cell gene expression data from normal and Alzheimer's disease-affected subjects.

**Results:**

We identified six co-expressed gene modules, each of which represented a biological process perturbed in Alzheimer's disease. Alzheimer's disease-related genes, such as *APOE*, *A2M*, *PON2 *and *MAP4*, and cardiovascular disease-associated genes, including *COMT*, *CBS *and *WNK1*, all congregated in a single module. Some of the disease-related genes were hub genes while many of them were directly connected to one or more hub genes. Further investigation of this disease-associated module revealed *cis*-regulatory elements that match to the binding sites of transcription factors involved in Alzheimer's disease and cardiovascular disease.

**Conclusion:**

Our results show the extensive links between Alzheimer's disease and cardiovascular disease at the co-expression and co-regulation levels, providing further evidence for the hypothesis that cardiovascular disease and Alzheimer's disease are linked. Our results support the notion that diseases in which the same set of biochemical pathways are affected may tend to co-occur with each other.

## Abstract 2

Analysis of microarray data reveals extensive links between Alzheimer’s disease and cardiovascular diseases.

## Background

Late-onset Alzheimer's disease (AD) is a complex progressive neurodegenerative disorder of the brain and is the most common form of dementia. Due to its polygenic nature, AD is believed to be caused not by defects in single genes, but rather by variations in a large number of genes and their complex interactions that ultimately contribute to the broad spectrum of disease phenotypes. Similar to other neurodegenerative diseases, AD has not yielded to conventional strategies for elucidating the genetic mechanisms and genetic risk factors. Therefore, a systems biology approach, such as the one that was successfully employed by Chen and colleagues [1], is an effective alternative for analyzing complex diseases.

**Figure 1:**
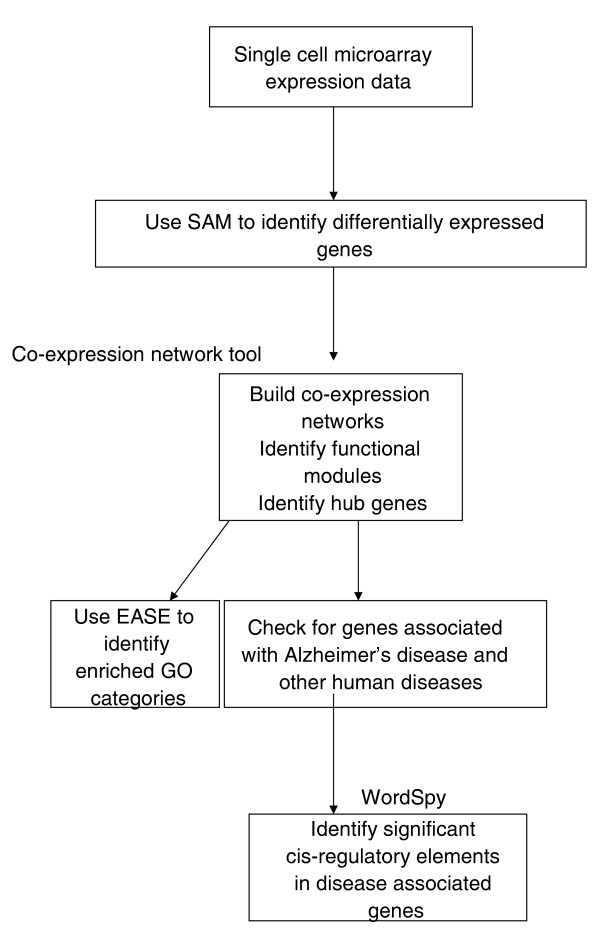
Steps taken to analyze Alzheimer's disease using laser capture microdissected microarray data. Sequence of steps taken to analyze incipient Alzheimer's disease from single cell expression data. We apply co-expression network analysis, EASE and WordSpy (motif finding method) in an integrated manner to study Alzheimer's disease and reveal connections to other conditions such as cardiovascular diseases and diabetes.

Most studies on AD first select a set of differentially expressed genes on which further analysis is performed. However, comparing lists of genes from various AD studies is not efficient without new methods being developed, which sometimes can become data specific. Therefore, organizing genes into modules or a modular approach that is based on criteria such as co-expression or co-regulation helps in comparing results across studies and obtaining a global overview of the disease pathogenesis. In this paper, we perform a transcriptome-based study by combining the analysis of co-expressed gene networks and the identification of functional modules and *cis*-regulatory elements in differentially expressed genes to elucidate the biological processes involved in AD [2-4]. We first construct modules of highly correlated genes (that is, those with high similarity in their expression profiles), and then identify statistically significant regulatory *cis*-elements (motifs) present in the genes. The analysis follows the procedure shown in Figure 1.

The present work unveiled 1,663 genes that are differentially expressed in AD. A co-expression network method [2,3] was applied to these genes, resulting in 6 modules of co-expressed genes with each module representing key biological processes perturbed in AD. Within the 6 modules, we identified 107 highly connected ('hub') genes. Functional annotation of these genes based on their association to human diseases resulted in the identification of 18 disease-related cardiovascular diseases (CVDs), AD/neurodegenerative diseases, stroke and diabetes) transcripts aggregating in one module (referred to as the disease associated module). While some of these 18 genes were hub genes, many of them directly connected to one or more hub genes. Furthermore, a genome-wide motif analysis [4] of the genes in the disease-associated module revealed several *cis*-regulatory elements that matched to the binding sites of transcription factors involved in diseases that are known to co-occur with AD. The final result was a set of co-expressed and co-regulated modules describing the higher level characteristics linking AD and CVDs.

Recently, Miller *et al*. [5] used a systems biology approach to identify the commonalities between AD and ageing. Our work is significantly different from that by Miller *et al*. as we use a different co-expression network building method to generate modules of co-expressed genes and then identify *cis*-regulatory motifs within a module. Such a combination of approaches has not been previously applied to study AD. Our co-expression network method [2,3] is a spectral algorithm that was designed to optimize a modularity function and automatically identify the appropriate number of modules. The *cis*-regulatory elements discovered in the promoter regions of disease related genes provide further insights into the possible transcriptional regulation of the genes involved in AD and their connection to CVDs, stroke and diabetes. Moreover, the single cell dataset [6] used in this study is less noisy compared to the mixed cell microarray data that were analyzed by Miller *et al*. Additionally, the single cell expression data are from the entorhinal cortex, a region of the brain known to be the germinal site of AD and, therefore, represent the early stage of AD (incipient AD). Most importantly, unlike multiple studies comparing AD and ageing [5,7,8], to the best of our knowledge, our study is the first that has identified links between CVDs, AD/neurodegenerative diseases and diabetes using a transcriptome-based systems biology approach. However, despite the differences in objectives, data and methods in the study by Miller *et al*. and in our study, there was a significant overlap in the results obtained. This indicates that the results reported here represent phenomena that are generalizable. We have established interesting links between the two studies, thereby highlighting the commonalities between AD, ageing, and CVDs. We believe that analyses such as ours and that by Miller *et al*. are the pieces of a puzzle that illustrates the underlying mechanisms involved in AD and the manner in which AD links to other conditions/diseases.

## Results and discussion

**Figure 2:**
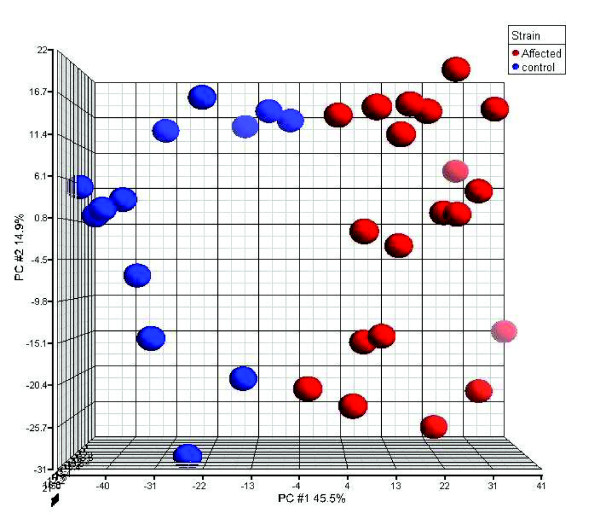
Unsupervised classification by principal component analysis. Principal component analysis was used to classify the 33 samples. The blue spheres refer to controls and the red correspond to affected subjects. This demonstrated that the samples were distinguishable based on the expression profiles of 1,663 differentially expressed genes.

Significance analysis of microarrays (SAM) [9] identified 1,663 differentially expressed genes between AD samples and controls at a false discovery rate of 0.1% (see Materials and methods). The enriched biological processes for 1,663 genes are shown in Additional data file 1. Many processes known to be affected in AD were enriched in the list of 1,663 transcripts. Principal components analysis [10] is an unsupervised classification method in which the data are segregated into classes. When principal components analysis was applied to a matrix consisting of the expression of 1,663 differentially expressed genes and 33 subjects (10 normal and 20 AD affected), an optimal separation of subjects into two groups was observed (Figure 2). The axes in Figure 2 correspond to the principal components (PCs), with the first PC accounting for 45.5% of the variance and the second PC accounting for 14.9% of the variance. This demonstrated that the samples are distinguishable based on the expression profiles of these 1,663 genes. This implies that the samples in this dataset are well characterized and the information content in these differentially expressed genes is high.

### Modular organization of significant genes via co-expression networks

**Figure 3:**
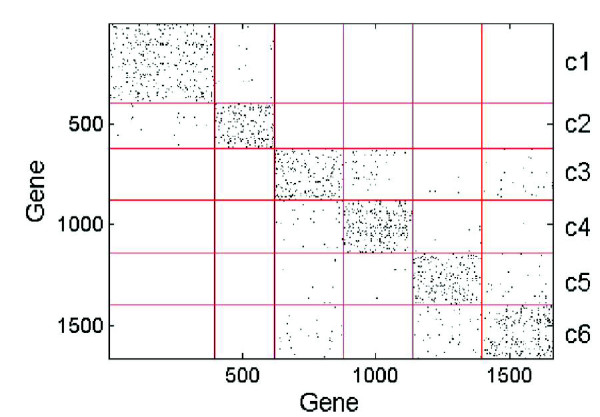
Adjacency matrix of co-expression network. The adjacency matrix representation of the co-expression network. Modules are labeled c1, c2, c3, c4, c5 and c6. The dots refer to the intra- and inter-module edges between the genes. The graphical representation of this matrix is in Additional data file 4.

**Figure 4:**
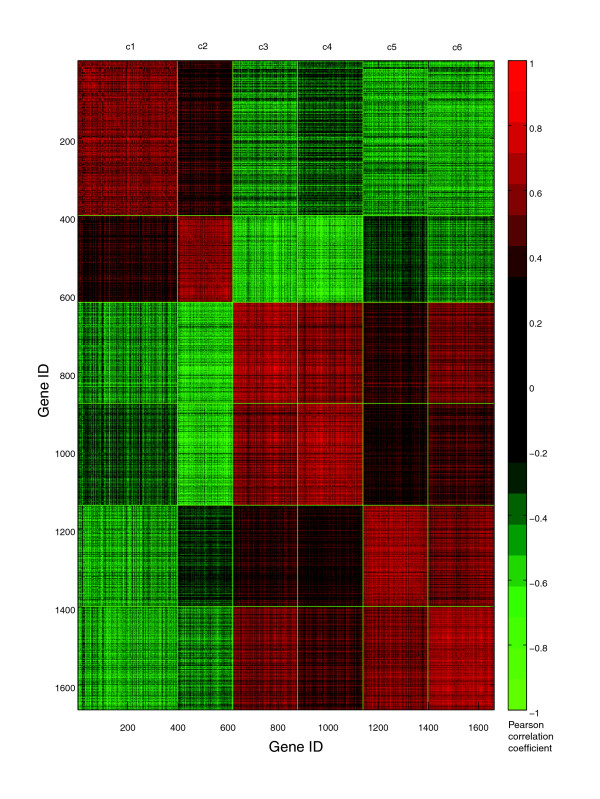
Pearson correlation coefficient between 1,663 genes. This figure shows the strength of correlation between pairs of genes. The genes are organized by modules - c1, c2, c3, c4, c5 and c6. The top leftmost red block on the diagonal corresponds to module c1 and the bottom rightmost red block on the same diagonal refers to module c6. Modules c1 and c2 contain upregulated genes and modules c3 through c6 comprise downregulated genes.

The co-expression network method (CoExp) [2,3] was applied to the set of 1,663 genes and resulted in 6 clusters/modules (see Materials and methods; a figure showing the entire network and modules is provided in Additional data file 4). Figure 3 shows the adjacency matrix of the co-expression network and Figure 4 illustrates the Pearson correlation coefficient (degree of similarity) between the 1,663 genes organized into modules. The effect of CoExp applied to all 15,827 genes (that is, no differentially expressed gene selection performed) is shown in Additional data file 5.

The two big red blocks of genes in Figure 4 represent two groups of anti-correlated expression patterns. The upper red block refers to modules 1 and 2, while the lower red block represents modules 3, 4, 5 and 6. Transcripts in modules 3, 4, 5 and 6 were downregulated and those in modules 1 and 2 were upregulated. Modules 1 and 2 contain transcripts involved in cell differentiation, neuron development, immune response, stress response, and so on, while the other modules consist of genes involved in negative regulation of metabolism, protein transport, sodium ion transport, and so on. Table 1 shows the top enriched Gene Ontology biological processes (*p *< 0.05) in all six modules.

**Table 1:** Top Gene Ontology biological processes in each module

Module	Activity	Ease score
Module 1	Protein biosynthesis	7.14E-06
	Cell development	2.37E-05
	Cell differentiation	4.88E-05
	Macromolecule biosynthesis	8.56E-05
	Cellular nerve ensheathment	1.11E-04
	Neuron development	2.22E-04
	Regulation of action potential	4.37E-04
		
Module 2	Response to other organism	0.004
	Immune response	0.014
	Defense response	0.020
	Response to stress	0.029
	Protein kinase cascade	0.030
	Integrin-mediated signalling pathway	0.030
	Myeloid cell differentiation	0.040
	JAK-STAT cascade	0.042
		
Module 3	Homophilic cell adhesion	2.58E-11
	Cell-cell adhesion	2.74E-09
	Nervous system development	3.44E-09
	Ion transport	0.007
	Gamma-aminobutyric acid signalling pathway	0.009
	Secretory pathway	0.019
	Small GTPase mediated signal transduction	0.028
	Sodium ion transport	0.036
		
Module 4	Cellular physiological process	6.91E-05
	Transcription from RNA polymerase II promoter	0.008
	Protein transport	0.014
	Post-chaperonin tubulin folding pathway	0.019
	Ubiquitin cycle	0.037
		
Module 5	Negative regulation of metabolism	0.011
	Actin filament depolymerization	0.025
	Barbed-end actin filament capping	0.025
	Negative regulation of actin filament depolymerization	0.025
	Negative regulation of protein metabolism	0.025
		
Module 6	Protein transport	0.008
	Cell organization and biogenesis	0.011
	Membrane fusion	0.028
	RNA processing	0.029
	RNA splicing	0.042

Statistically significant (*p *< 0.05) biological processes present in each of the six modules of the co-expression network.

As can be noted from Table 1, many processes linked to AD, such as immune response, inflammatory response, cell development and differentiation (due to a large number of cancer related genes), and so on are upregulated in incipient AD [11,12]. Processes related to actin are downregulated in AD [13]. Table 2 shows the significant Kyoto Encyclopedia of Genes and Genomes (KEGG) pathways represented by the genes in each module. Although there was no over-represented KEGG pathway in module 5, several genes involved in the negative regulation of metabolism, actin filament depolymerization, glucose metabolism, and lipid biosynthesis were present. Modules 2, 3, 4, 5 and 6 represent processes previously associated with AD in multiple studies [11-13]. Module 5 contains processes related to glucose metabolism and recent work has shown decreased expression of energy metabolism genes [14]. Our results further confirm this observation. Based on the results obtained thus far, each module is representative of some biological processes: module 1 represents protein synthesis; module 2 is linked to phospholipid degradation; module 3 is associated with signaling systems; module 4 represents neuron development; and modules 5 and 6 are associated with metabolism.

**Table 2:** Statistically significant KEGG pathways

Module	KEGG pathway	Ease score
Module 1	Ribosome	8.16E-07
	Translation	3.41E-14
		
Module 2	Phospholipid degradation	0.013
		
Module 3	Signal transduction	0.002
	Phosphatidylinositol signaling system	0.005
		
Module 4	Neuron development	2.22E-04
		
Module 6	Nucleotide metabolism	0.036

Statistically significant (*p *< 0.05) KEGG pathways present in the modules of the co-expression network.

The modular organization of genes led to the following investigative steps: the identification of genes associated with human diseases; the identification of hub/highly connected genes; the examination of the expression level of *brain derived neurotrophic factor *(*BDNF*) in the AD subjects; and the identification of *cis*-regulatory elements from the promoters of genes.

### Module 1 is associated with cardiovascular diseases and diabetes

EASE [15] uses the Genetic Association Database [16] and Online Mendelian Inheritance in Man to determine the association of genes with various diseases/conditions [17-19] (see Materials and methods). When EASE was used to perform functional annotation clustering based on the genes' association with human disorders/diseases, module 1 contained 18 disease-associated genes (Table 3). This prompted an in-depth examination of module 1 for our downstream analysis. Modules 2-6 did not have a significant enrichment for any human disease.

**Table 3:** Functional annotation clustering by disease of genes

Disease/condition	Genes
Neurodegeneration	*VWF*, *A2M*, *APOE*, *FTL*, *PON2*, *COMT*, *MAP4*, *TF*, *SERPINA3*, *ATP1A2*, *AGT*
Myocardial infarction	*A2M*, *APOE*, *PON2*, *SERPINA3*
Alzheimer's disease	*A2M*, *APOE*, *SERPINA3*, *PON2*
Cardiovascular	*VWF*, *A2M*, *APOE*, *PON2*, *COMT*, *WNK1*, *CBS*, *SERPINA3*, *TIMP1*
Coronary artery disease	*APOE*, *PON2*, *COMT*, *SERPINA3*

Type 2 diabetes	*VWF*, *A2M*, *APOE*, *PCBD2*, *HLA-DQB1(HLA-DQB2)*, *TIMP3*, *SLC2A1*, *AGT*

Functional annotation clustering of genes in module 1 based on their association to human conditions/diseases.

These results provide new evidence supporting the hypothesis that there may be a strong association between CVD and the incidence of AD [20-22]. There also has been a growing body of evidence for a link between AD and diabetes [23-25], with many research groups and news articles reporting that AD may be another form of diabetes. While there are many transcripts in Table 3 common to the different conditions, there are a few that are unique to a specific disease/condition, such as those encoding *kinase deficient protein *(*WNK1*), *timp metallopeptidase inhibitor 1 *(*TIMP1*) and *cystathionine-beta-synthase *(*CBS*), which are specific to CVD. *Pterin-4 alpha-carbinolamine dehydratase/dimerization cofactor of hepatocyte nuclear factor 1 alpha *(*tcf1*) *2 *(or *PCBD2*), *timp metallopeptidase inhibitor 3 *(*TIMP3*), *solute carrier family 2 member 1 *(*SLC2A1*) and major *histocompatibility complex, class II, dq beta 1 *(*HLA-DQB1*) are specific to diabetes. *Von willebrand factor *(*VWF*), *alpha-2-macroglobulin *(*A2M*), *apolipoprotein e *(*APOE*), *paraoxonase 2 *(*PON2*), and *serpin peptidase inhibitor, clade a *(alpha-1 antiproteinase, antitrypsin), member 3 (*SERPINA3*) are common to most of the conditions. Archacki and colleagues have reported a list of 56 genes that are associated with coronary artery disease [26]. Many genes from this list were also present in our list of 1,663 genes and present in module 1 (data not shown).

The hypothesis behind co-expression network analysis is that genes that are co-expressed are also co-regulated. Therefore, since the genes specific to certain diseases and those that are common to all the diseases all resided in the same module, they may be co-regulated. This could be the reason for the clustering of these conditions in epidemiological studies. Furthermore, as there are many transcripts common to these diseases/conditions, it is plausible that similar/common biochemical pathways are active in these seemingly different conditions. Common pathogenetic mechanisms in AD and CVD can suggest a causal link between CVD and AD [21,22], a hypothesis that is still controversial and under a lot of debate.

Transcripts in the modules are linked to each other based on their expression similarity. 'Hub genes' are highly connected nodes/transcripts in the network and are likely to play important roles in biological processes. Hub genes tend to be conserved across species and, hence, make excellent candidates for disease association studies in humans [27].

We defined hub genes to be those with 40 or more links/connections. Please refer to Additional data file 6 for the estimation of hub genes. We identified 107 hub genes. The complete list of hub genes, their module locations, and the number of links is in Additional data file 2. The hub genes included those encoding general *transcription factor iiic, polypeptide 1, alpha *220 kda (*GTF3C1*), which is involved in RNA polymerase III-mediated transcription, *microtubule-associated protein 4 *(*MAP4*), which promotes microtubule stability and affects cell growth [28], and *proprotein convertase subtilisin/kexin type 2 *(*PC2*), which is responsible for the processing of neuropeptide precursors. Some of these hub genes - *PC2*, *paraoxonase 2 *(*PON2*) and *peroxiredoxin 6 *(*PRDX6*) - have been implicated in late-onset AD [29-31].

**Figure 5:**
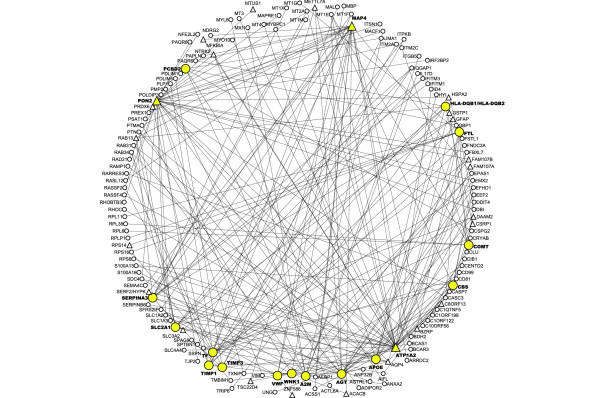
Sub-network in module 1 illustrating the 18 disease associated genes and their connections. This sub-network shows the 18 disease associated genes (colored yellow) and the genes that they are connected to within module 1. The hub genes are represented as triangle nodes. Disease genes *MAP4*, *PON2 *and *ATP1A2 *were also hub genes. Only the hub genes that connect to disease genes are shown here. Module 1 consists of 22 hub genes in total.

Since module 1 has the disease associated genes, the hub genes in this module may provide new information regarding AD, CVD and diabetes. We identified 22 hub genes with a number of links ranging from 42 to 63 in module 1 (for the complete list of the 22 hub genes, see Additional data file 2). The total number of hub genes in each module along with the minimum and maximum number of links is shown in Table 4. Module 1 had the maximum number of hub genes. The transcript with the largest number of links in module 1 is *MAP4*, with 63 connections. *MAP4 *is directly linked to other disease/condition associated genes such as *VWF *and *WNK1*. Increased expression of *semaphorin 3b *(*SEMA3B*; semaphorin pathway) inhibits axonal elongation [32] and has been implicated in AD [32]. *MAP4 *is also connected to *SEMA3B*. Table 5 shows the number of links of the disease associated genes and the number of hub genes they are linked with. Figure 5 is a sub-network in module 1 that shows the disease-associated genes and all their links within module1. Although not all the disease-associated genes were hub genes, most of them were directly linked to one or more hub genes, which implies that they may play a key role via hub genes.

**Table 4:** Hub genes

Module	Number of hubs	Range of links
Module 1	22	42-63
Module 2	17	41-56
Module 3	15	40-68
Module 4	14	40-65
Module 5	20	40-73
Module 6	19	40-81

Number of hub genes and their range of connections/links in each module.

**Table 5:** Number of links of the 18 disease-associated genes

Gene	Number of links	Number of hub genes it is connected to
*VWF*	16	2
*A2M*	17	3
*APOE*	18	3
*FTL*	18	3
*PON2*	51	8
*COMT*	17	0
*MAP4*	63	5
*TF*	16	3
*SERPINA3*	18	3
*ATP1A2*	45	7
*AGT*	27	5
*TIMP1*	14	3
*WNK1*	17	2
*CBS*	16	3
*PCBD2*	16	0
*HLA-DQB1/HLA-DQB1*	15	2
*SLC2A1*	14	4
*TIMP3*	14	0

Number of links of the 18 disease associated genes from module 1 and the number of connections they have with other hub genes.

*PON2*, *MAP4 *and *atpase Na+/K+ transporting, alpha 2 (+) polypeptide (ATP1A2) *are encoded by disease-associated genes that are also hub genes. The overexpression of *MAP4 *results in the inhibition of organelle motility and trafficking [33] and can also lead to changes in cell growth [28]. *ATP1A2 *is a subunit of an integral membrane protein that is responsible for establishing and maintaining the electrochemical gradients of sodium and potassium ions across the plasma membrane [34]. These gradients are essential for osmoregulation, for sodium-coupled transport of a variety of molecules, and for electrical excitability of nerve and muscle [34]. While the downregulation of *ATP1A2 *has been linked to migraine-related conditions [35], the effects of its upregulation have not been documented. PON2 has been implicated in AD [30] and CVDs (Table 3).

### Decreased levels of *brain-derived neurotrophic factor*

*BDNF *is well known for its trophic functions and has been implicated in synaptic modulation, and the induction of long-term potentiation [36,37]. Increased levels of *BDNF *are necessary for the survival of neurons. Decreased levels of *BDNF *have been linked to AD and depression [38-40]. Recently, low levels of *BDNF *has also been associated with diabetes [41].

*BDNF *goes through post-translational modification, that is, it is converted into mature *BDNF*, by plasminogen [42]. The *neurotrophic tyrosine kinase receptor type 2 (NTRK2/TrkB) *is a receptor for *BDNF *[43].

*BDNF *was not present in our list of 1,663 significant genes. However, *TrkB *and *serpin peptidase inhibitor, clade e (nexin, plasminogen activator inhibitor type 1), member 2 (SERPINE2) *were present in the set of 1,663 genes and located in module 1. *Plasminogen activator inhibitor type 1 (PAI-1) *proteins inhibit plasminogen activators [44]. Therefore, if the level of *PAI-1 *is high in the AD affected samples, plasminogen activators are being inhibited, resulting in decreased levels of mature *BDNF*. Interestingly, the expression levels of *TrkB *and *PAI-1 *were elevated in the AD samples. However, *TrkB *is downregulated following the binding of *BDNF *[45]. Therefore, due to an increased level of *PAI-1*, mature *BDNF *could not be produced, which in turn could not bind to *TrkB*. By this reasoning, it can be concluded that high levels of *TrkB *and *PAI-1 *imply decreased levels of *BDNF*, which is detrimental for the survival of neuronal populations. This probably leads to neuronal death in this cohort of AD affected subjects.

In order to verify our conclusion regarding the expression level of *BDNF *in the AD patients in our dataset, we examined the expression level of *BDNF *in the controls and AD affected samples. We found *BDNF *to be decreased by 1.07 in the AD affected samples. *BDNF *was not selected to be a significant gene, probably because it had a small difference in the expression between controls and affected samples. Microarrays are not sensitive enough to detect genes with low expression levels, especially when the difference in expression is small (which can be expected in subjects with incipient AD) [46-49]. The fact that the selected significant genes, such as *TrkB *and *SERPINE2*, could lead to the correct conclusion regarding the level of *BDNF *expression in AD affected samples highlights the merits of this kind of analysis of the transcriptome when handling genes with low expression levels. Although modules 1 and 2 have upregulated genes, genes associated with *BDNF *are located only in module 1. This further emphasizes the importance of module 1.

### Comparison to the study by Miller *et al*. on ageing and AD

Miller *et al*. [5] identified 558 transcripts that were common to AD and ageing. We found more overlapping genes between our study and their study than expected by chance (*p *= 3.3 × 10^-10^). There were 94 genes overlapping between 1,663 significant genes from our study and 558 genes identified by Miller *et al*. Of these 94 genes, 48 were present in module 1 (greater than expected by chance; *p *= 9.2 × 10^-10^). This indicates that module 1 contains the majority of genes that have been linked to ageing and AD. Of the 48 genes that overlapped between 558 AD-ageing common genes and genes in module 1, *WNK1 *and *MAP4 *were present.

Furthermore, 9 genes (*DAAM2*, *EPM2AIP1*, *GFAP*, *GORASP2*, *MAP4*, *NFKBIA*, *PRDX6*, *TSC22D4 *and *UBE2D2*) overlapped between 558 AD-ageing genes and the 107 hub genes identified in our study, 5 of which resided in module 1. These results further highlight the significance of module 1 and it can be concluded that module 1 represents common biochemical pathways that may be affected in all AD, ageing, and CVD.

### *Cis*-regulatory elements and co-regulated genes

*Cis*-regulatory elements/motifs are regulatory elements in the promoter region of genes to which transcription factors bind, thus regulating transcription. If a group of genes shares the same *cis*-regulatory motif, then the transcription factor that binds to the motif may regulate the group of genes. Co-expressed modules represent genes that may be co-expressed in the cell and be a part of the same biochemical pathways. From our analyses thus far, we concluded that the genes contained in module 1 is of great importance. Therefore, we used WordSpy [4] to identify the *cis*-regulatory elements/motifs that may be enriched in the upstream promoter sequences of the genes in module 1 (see Materials and methods). The group of genes in module 1 that shares a motif will be a set that is co-expressed and coregulated.

The complete set of *cis*-regulatory elements enriched in module 1 is in Additional data file 3. A total of 89 motifs were enriched in module 1 with a *p*-value < 0.001, and their target genes were co-expressed with an average correlation coefficient >0.4 and *Z*-score >2 (see Materials and methods). Of the 89 motifs, 36 matched to 26 known transcription factor binding sites (TFBS) in JASPAR [50] with a matching score ≥0.8 (Table 6). Table 6 shows the number of genes within module 1 whose promoter region contains a motif that matched to the TFBS of a known transcription factor.

**Table 6:** Twenty-six transcription factors with known functions whose *cis*-regulatory elements were identified in the genes in the co-expression network

Transcription factors	Number of target genes
ABI4	9
Arnt-Ahr	93
ARR10	6
Broad-complex 3	10
CEBP	20
Gfi	8
HAND1-TCF3	279
Mycn	11
Myf	8
Prx2/PRDX2	17
RELA, REL	10
RUNX1	4
Snail	49
SP1	47
TBP	6
E74A	16
ELK1	16
SPIB	16
Hunchback	6
MAX	11
USF1	11
ZNF42 5-13	27
NFIL3	5
Agamous	8
GAMYB	6

The 26 transcription factors and the number of target genes in module 1 that have a motif in their promoters that match to the binding sites of the known transcription factor.

Transcription factors such as *growth factor independent (Gfi), peroxiredoxin 2 (Prx2/PRDX2), SP1, CAAT-enhancer binding protein (C/EBP), RelA (p65), runt box 1 (Runx1), ELK-1, upstream stimulatory factor 1 (USF1), Rel*, and *TATA box binding protein (TBP) *have been implicated in neurodegenerative diseases (such as AD, Parkinson's, and Schizophrenia) [51-64], diabetes [65], stroke and CVDs [66,67]. There are 139 genes in module 1 that contain motifs that matched the TFBS of the known transcription factors associated with these diseases.

*Arnt-Ahr *dimer transcription factor activates genes crucial in the response to hypoxia and hypoglycaemia [68,69]. Hypoglycaemia and hypoxia have been known to play pathophysiological roles in the complications of diabetes and AD [70-73]. It is well known that hypoxia has major effects on the cardiovascular system [74]. In light of such knowledge, it comes as no surprise that a large number of genes have *cis*-regulatory motifs that match the binding site of the Arnt-Ahr transcription factor.

*Hand1-TCF3 *and *TAL1-TCF3 *are components of the *basic-helix-loop-helix (bHLH) *complexes. *bHLH *transcription factors are important in development [75,76]. An extremely high number of genes were mapped to *Hand1-TCF3 *since cell development and differentiation is upregulated in AD [11,12].

In summary, the fact that transcription factors that participate in other human conditions have their binding motifs enriched in the set of significant genes associated with AD adds significance to the hypothesis that many biochemical pathways common to AD and CVD are active, resulting in these diseases/conditions co-occurring.

## Conclusion

In this study, we present an integrative systems biology approach to study a complex disease such as AD. Along with identifying modules that illuminate higher-order properties of the transcriptome, we identified a module that contained many genes known to play prominent roles in CVDs and AD. We believe that this module highlights important pathophysiological properties that connect AD, CVD and ageing. We identified several *cis*-regulatory elements, some of which mapped to the binding sites of known transcription factors involved in neurodegenerative and CVDs as well as diabetes and stroke. Furthermore, since microarrays are not sensitive to genes with very slight differences in expression from controls, we illustrate how other genes can be used to deduce the expression difference of such genes. This is especially critical while comparing groups that are very similar to each other.

Although we highlight the contributions of a new module and network building method to the field of AD, this paper also illustrated the commonalities between the study by Miller *et al*. [5] and our study in spite of the differences in methodology and data. This suggests the reproducible and generalizable quality of the results based on gene expression data from well characterized samples. Additionally, a modular approach, where genes are organized into modules based on co-expression or co-regulation, is an efficient method for studying human diseases and comparing results from multiple studies.

The link between CVDs, diabetes and AD is a topic of growing interest. The presence of perturbed genes and *cis*-regulatory elements related to CVDs and AD in a single module provides strong evidence to the hypotheses connecting these two conditions. Interestingly, this module also contained the maximum number of genes (and hub genes) related to ageing. Our results support the notion that diseases in which the same set of biochemical pathways are affected may tend to co-occur with each other. This could be the reason why CVDs and/or diabetes co-occur with AD.

Small sample sizes are typical of clinical studies, especially those involving human samples. The largest AD gene expression study at the time of writing included 33 samples (the dataset analyzed in this paper). Since the results presented here may be specific to the dataset, we are in the process of extending our analysis to larger datasets. A more robust approach to studying AD would be to obtain well characterized large cohorts that are followed longitudinally for the best chance of success. A comprehensive analysis incorporating AD and CVD/diabetes patients along with information about their disease progression will shed more light onto the pathophysiology of, and the link between, AD and CVDs.

## Materials and methods

### Data

Pathologically, AD is characterized by the presence of neurofibrillary tangles in the neurons. The dataset of Dunckley *et al*. [6] consists of 13 normal controls (Braak stages 0-II; average age 80.1 years) and 20 AD affected (Braak stages III-IV; average age 84.7 years) samples obtained by laser capture microdissection from the entorhinal cortex. Braak stages III-IV are considered 'incipient' AD [77,78]. In this dataset, 1,000 neurons were collected from each of the 33 samples via laser capture microdissection.

Data were normalized using gcRMA [79]. Probesets were mapped to genes using DAVID [34]. Probesets that did not map to any gene and those that mapped to hypothetical proteins, at the time of writing this manuscript, were removed. When multiple probesets mapped to the same gene, only the probeset with the highest mean was selected. This preprocessing resulted in 15,827 genes/transcripts. Differentially expressed genes were identified using the two-class SAM procedure [9]. SAM is open-source software that uses a modified t-statistics approach to identify differentially expressed genes. ISI citation search [80] indicates that SAM is a highly popular method used for microarray analysis (over 2,000 citations of the original publication in April 2001, as of July 9, 2008).

### Construction of co-expression networks and identification of functional modules

We used a network-based approach to identify modular structures/clusters embedded in microarray gene expression data. The CoExp [2,3] method constructs co-expression networks from microarray data and then uses a spectral based clustering method to identify subgraphs within the network. Nodes in the network correspond to genes and edges represent expression similarities between genes. The motivation is that genes involved in the same functional pathway are directly connected to each other or linked via short paths. After network creation, the nodes are clustered into dense subgraphs.

To create a network from gene expression data, pairwise expression similarity between a pair of genes was measured. In this study, we used the Pearson correlation coefficient for the similarity measure. For two genes to be considered as co-expressed, their expression profiles needed to satisfy at least one of the following conditions: their correlation coefficient is higher than 0.3, and one gene is ranked as the top-*k *most correlated gene of the other; the correlation coefficient between them is higher than 0.9 and one gene is within the top 50 most correlated gene of the other. The parameter *k *was determined automatically and in conjunction with the Qcut algorithm (discussed below), such that when *k *increased, the number of modules of co-expressed genes remained unchanged. The rationale behind using *k *best neighbors instead of a cut-off threshold on gene expression similarity for creating a network has been discussed in [2]. For the co-expression network generated with differentially expressed genes in this study, *k *= 14.

In order to identify dense subgraphs/modules in the co-expression network, we applied a community discovery algorithm - Qcut, developed by Ruan and Zhang [3]. Compared to other clustering or graph partitioning algorithms, Qcut has the advantage that it does not require a user-specified number of clusters/modules. It is a spectral based graph partitioning algorithm that optimizes the modular function proposed by Newman and Girvan [81] to automatically determine the appropriate number of modules [2,3]. Further evidence of its robustness can be found in [3,82].

EASE [15], a tool in DAVID, was used to identify overrepresented biological processes in each module as well as perform functional annotation clustering based on association to human diseases [34]. DAVID derives its disease associations from two main sources, Online Mendelian Inheritance in Man and the Genetic Association Database. These sources assign diseases to gene identifiers and then DAVID maps the diseases to the DAVID database through the gene identifiers. The most significant diseases associated with a set of genes are determined by term enrichment analysis using a modified Fisher Exact calculation [17-19].

### Identification of regulatory *cis*-elements

The interaction of transcription factors and *cis*-acting DNA elements determines the gene activity under various environmental conditions. Identifying functional TFBS, however, is not trivial, since they are usually short and degenerate, and are often located several hundred to thousand bases upstream of the translational starting sites. Here we combined several datasets and a whole-genome analysis method, WordSpy [4], to discover short DNA sequence motifs that are statistically enriched in the promoters of genes in the same co-expression module and are associated with gene co-expression.

We first downloaded the promoter sequences for human open reading frames from the DBTSS database [83]. Each promoter included 1,000 bp upstream and 200 bp downstream sequences relative to the transcription starting site, defined from full length cDNA data. From this dataset we extracted *n *sets of promoter sequences (referred to as experimental sets), where *n *is the number of co-expression modules. The *i*-th experimental set contains the promoter sequences of genes in the *i*-th co-expression module. The complete set of human gene promoters was used as the background set. We then applied WordSpy, a steganalysis-based genome-wide motif-finding method, on each experimental set to discover statistically significant *k*-mers (motifs; for *k *= 6, 7, 8, 9, 10) according to a generative model of the promoter sequences.

Each *k*-mer that was identified by WordSpy was then subjected to two filtering steps. In the first filtering step, motifs that are specifically enriched in the experimental set were selected. We counted the number of instances that a *k*-mer appeared in the experimental set (denoted by *x*) and in the background set (denoted by *b*). Then we computed the probability that we would expect by chance at least the same number of occurrences in the experimental set, given the number of occurrences in the background set. This probability is computed using the cumulative hyper-geometric distribution as:

$$P(x,b,N_{i},N)=\sum_{k=x}^{\min(N_{i},b)}\frac{\left(\begin{array}{c} N_{i}\\ k \end{array}\right)\left(\begin{array}{c} N-N_{i}\\ b-k \end{array}\right)}{\left(\begin{array}{c} N\\ b \end{array}\right)},$$

where *Ni *and *N *are the sizes of the *i*-th experimental set and the background set, respectively. We filtered out the *k*-mers that had a *p*-value ≥ 0.01.

The second filter is used to select motifs that are associated with strong and significant co-expression patterns. For each motif that passed the first filtering phase, we obtained a set of genes ('target set') in which each gene in this set contains the motif in its promoter region. We computed the average pair-wise Pearson correlation coefficients, denoted by *pcc*, from the expression profiles of the genes in the target set. Furthermore, we randomly sampled 100 control sets of genes from the background set that had the same size (that is, number of genes) as the target set, and computed the *pcc *of each control set. The mean and standard deviation (denoted by *mpcc *and *spcc*, respectively) of the *pcc *values for the control sets are then used to compute the *Z*-score of the *pcc *value for the target set as:

$$Zscore=\frac{pcc-mpcc}{spcc}$$

A motif is retained only if its *pcc *> 0.4, and its *Z*-score > 2.

Finally, the motifs that have passed both filters are compared to the known TFBS in the JASPAR database [50]. We pre-filtered the TFBSs in the database that have information content ≤6 bits, since these TFBSs are short and have high degeneracy and, hence, may match to some known motifs simply by chance. Then we computed the best un-gapped alignment between the motifs (*n*-mers) and the known binding sites (position specific weight matrices) using a metric called the information score, which is the metric used in Matlnspector [84] in the TRANSFAC suite. If the information score for a motif is ≥0.8, then it is considered as a motif matching to the binding site of a transcription factor.

## Abbreviations

AD: Alzheimer's disease; BDNF: brain-derived neurotrophic factor; CoExp: co-expression network method; CVD: cardiovascular disease; KEGG: Kyoto Encyclopedia of Genes and Genomes; MPCC: mean of the PCC values; PAI-1: plasminogen activator inhibitor type 1; PC: principal component; SAM: significance analysis of microarrays; SPCC: standard deviation of the PCC values; TFBS: transcription factor binding sites.

## Authors' contributions

WZ conceived of the research. MR and WZ designed the study. MR and JR carried out the computational analysis, and MR performed the biological analysis as well as coordinated the project. MR wrote the paper and WZ helped with the manuscript preparation. All authors read and approved the final manuscript.

## Additional data files

The following additional data are available with the online version of this paper. Additional data file 1 lists the enriched biological processes in the set of 1,663 genes (*p *< 0.05). Additional data file 2 shows the 107 hub genes with 40 or more connections and the clusters in which they reside. Additional data file 3 contains the 89 statistically significant motifs over-represented in module 1 along with their *p*-values and *Z*-scores. Additional data file 4 shows the graphical representation of the coexpression network with 1,663 differentially expressed genes. Additional data file 5 shows the adjacency matrix of the co-expression network analysis on 15,827 genes. Additional data file 6 illustrates the distribution of co-expression network links and estimation of hub genes.

## Supplementary Material

Additional data file 1 Enriched biological processes in the set of 1,663 genes (*p *< 0.05). Click here for file

Additional data file 2 The 107 hub genes with 40 or more connections and the clusters in which they reside. Click here for file

Additional data file 3 The 89 statistically significant motifs over-represented in module 1 along with their *p*-values and *Z*-scores. Click here for file

Additional data file 4 This co-expression network shows six modules. A node refers to a gene and the weight of an edge is the Pearson correlation coefficient between expression profiles of a pair of genes scaled to within [0,1]. The two large groups are two sets of genes with anti-correlated expression patterns. The smaller group contains two modules (1 and 2) and consists of upregulated genes while the larger group (modules 3-6) consists of downregulated genes. The length of each edge and the position of each node/module does not have any biological meaning and are arbitrarily chosen for proper visualization. Click here for file

Additional data file 5 The CoExp was applied to the entire set of 15,827 genes and resulted in 13 clusters. Clusters/modules are labeled 1-13 and are shown at the top. The dots refer to the intra- and inter-module edges between the genes. Cluster 1 contains all the 18 disease-associated genes and genes involved with BDNF. The co-expression network does not need differentially expressed genes and can be used on any set of genes selected by some criterion. However, most studies on AD first select a set of differentially expressed genes on which further analysis is performed. We extracted differentially expressed genes since our goal was to study the underlying mechanisms involved in late onset AD and compare our results with other AD studies. The non-differentially expressed genes bear little significance in revealing the underlying biological processes affected in AD. Click here for file

Additional data file 6 The graph plots the number of links for the differentially expressed genes within the co-expression network. The X-axis plots the genes (as gene ID) in ascending order of the number of links. Gene ID 1 refers to the first gene, gene ID 800 refers to the 800th gene. The Y-axis plots the number of links for each gene. The dashed line indicates the mean number of links, and the solid line indicates the hub gene cutoff. The average number of links = 22.06; median = 19; standard deviation = 9.32. Gene co-expression networks follow power-law distributions and are scale-free, small world networks. They are characterized by a small number of highly connected nodes. In order to find a conservatively small number of hub genes, we decided to use a cut-off value that is towards the right of the distribution. Threshold for the number of links for hub genes = Mean + 2 × Standard deviation = 40.7. Genes with a number of links ≥40 were considered hub genes. This approach resulted in 6.4% being hub genes in the entire network. Click here for file
