# Supplementary figures and images for: Variations in the transcriptome of Alzheimer's disease reveal molecular networks involved in cardiovascular diseases
Source: Genome Biol. 2008 Oct 8;9(10):R148. doi: 10.1186/gb-2008-9-10-r148 (PMC2760875; doi:10.1186/gb-2008-9-10-r148)

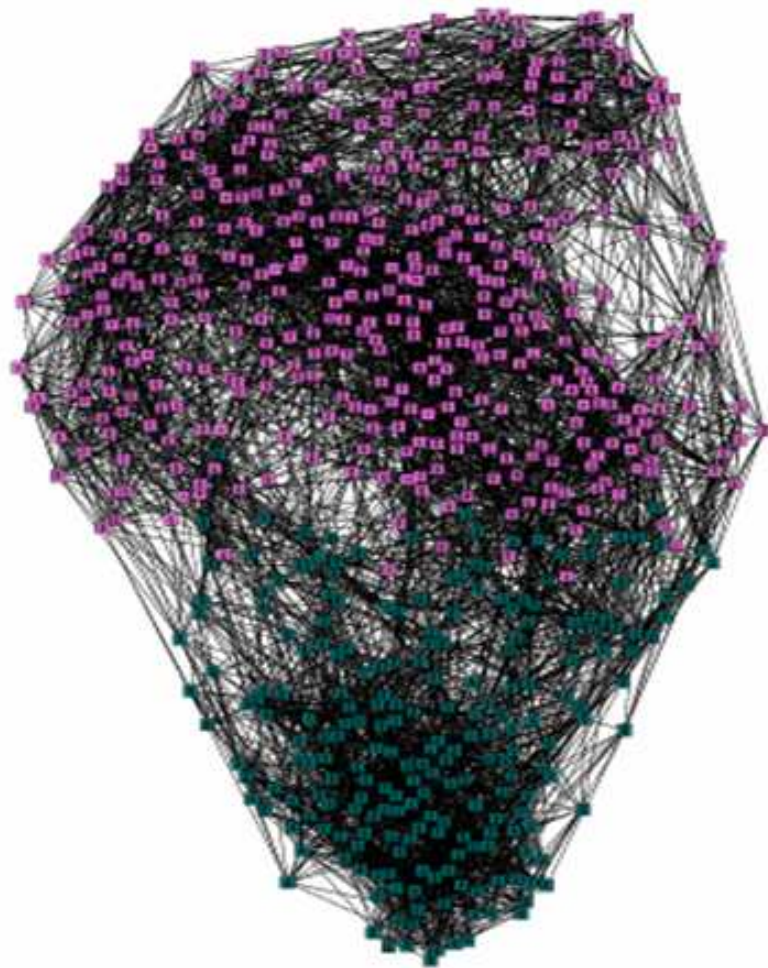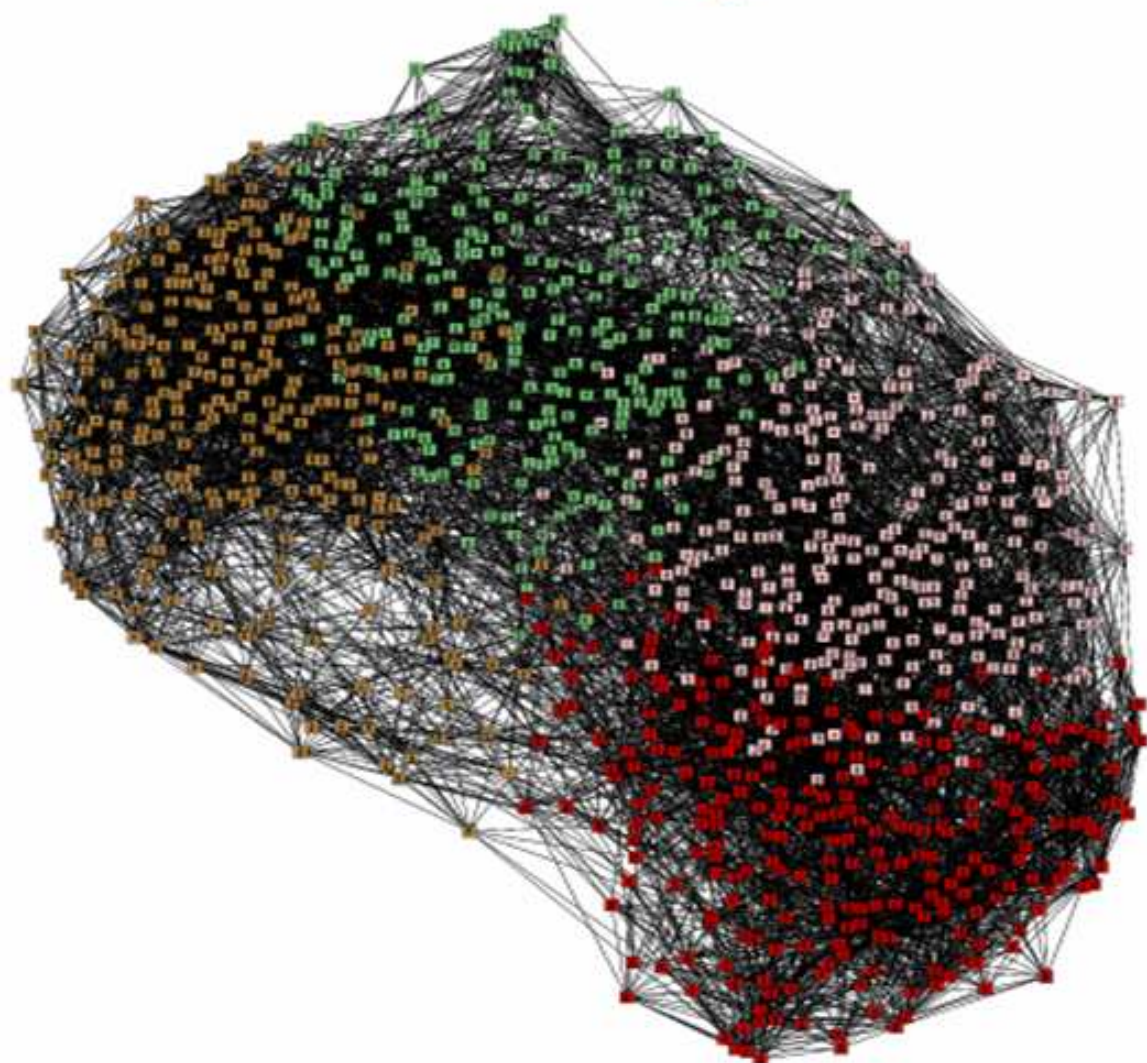

Supplement: Additional data file 4 — This co-expression network shows six modules. A node refers to a gene and the weight of an edge is the Pearson correlation coefficient between expression profiles of a pair of genes scaled to within [0,1]. The two large groups are two sets of genes with anti-correlated expression patterns. The smaller group contains two modules (1 and 2) and consists of upregulated genes while the larger group (modules 3-6) consists of downregulated genes. The length of each edge and the position of each node/module does not have any biological meaning and are arbitrarily chosen for proper visualization. [file gb-2008-9-10-r148-S4.pdf]

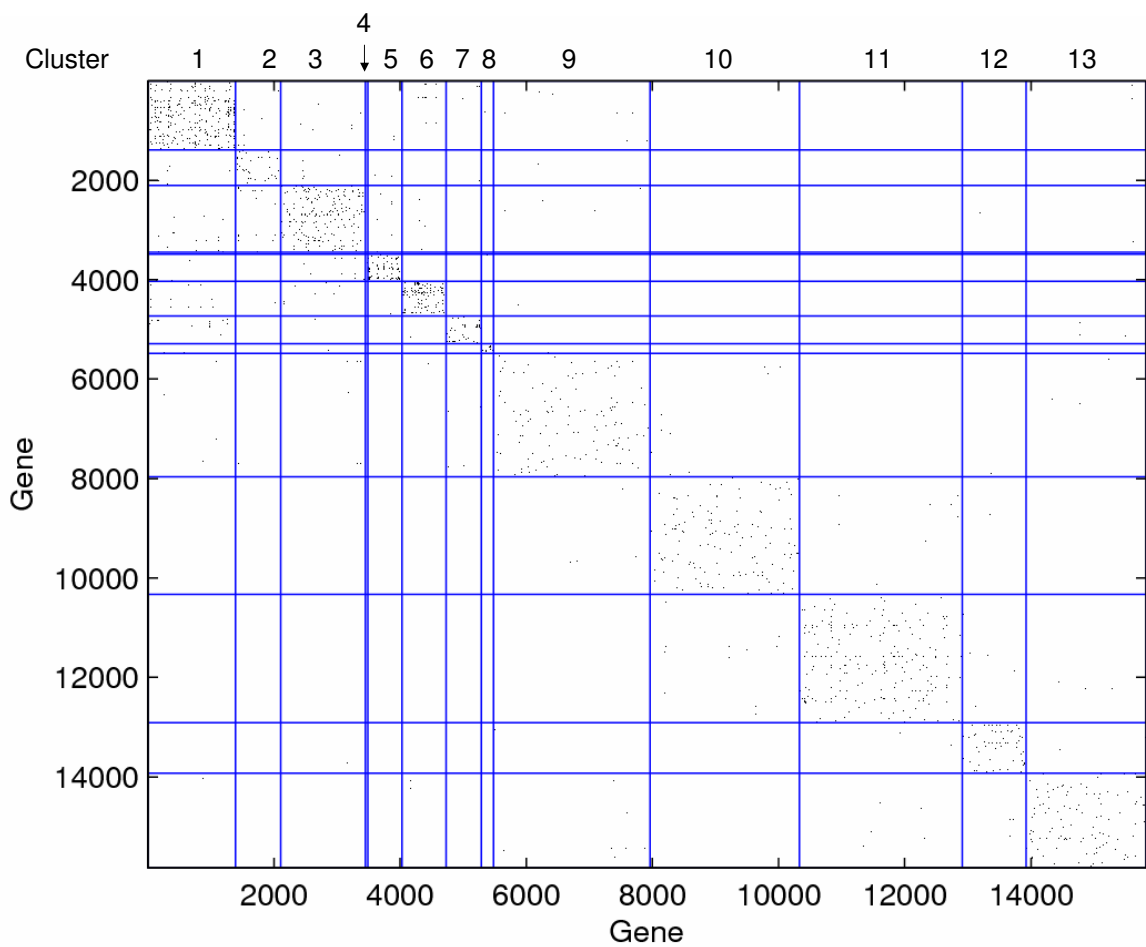

Supplement: Additional data file 5 — The CoExp was applied to the entire set of 15,827 genes and resulted in 13 clusters. Clusters/modules are labeled 1-13 and are shown at the top. The dots refer to the intra- and inter-module edges between the genes. Cluster 1 contains all the 18 disease-associated genes and genes involved with BDNF. The co-expression network does not need differentially expressed genes and can be used on any set of genes selected by some criterion. However, most studies on AD first select a set of differentially expressed genes on which further analysis is performed. We extracted differentially expressed genes since our goal was to study the underlying mechanisms involved in late onset AD and compare our results with other AD studies. The non-differentially expressed genes bear little significance in revealing the underlying biological processes affected in AD. [file gb-2008-9-10-r148-S5.pdf]

### Number of coexpression network connections per gene

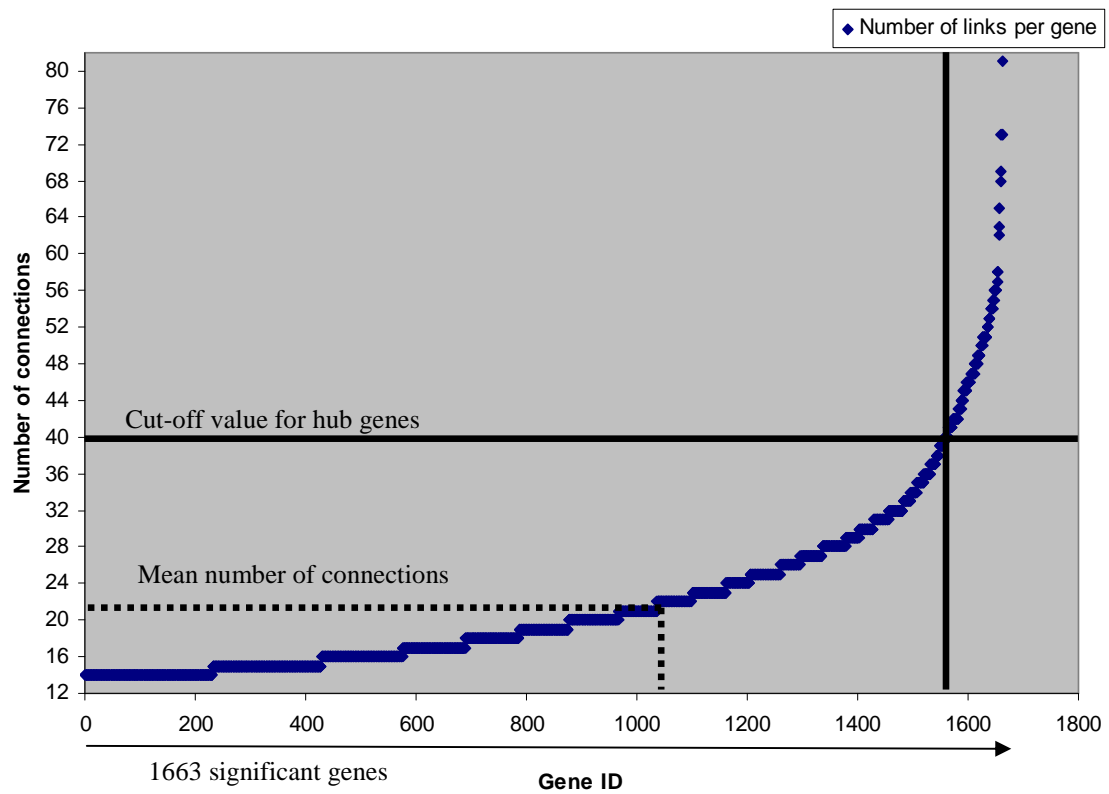

Supplement: Additional data file 6 — The graph plots the number of links for the differentially expressed genes within the co-expression network. The X-axis plots the genes (as gene ID) in ascending order of the number of links. Gene ID 1 refers to the first gene, gene ID 800 refers to the 800th gene. The Y-axis plots the number of links for each gene. The dashed line indicates the mean number of links, and the solid line indicates the hub gene cutoff. The average number of links = 22.06; median = 19; standard deviation = 9.32. Gene co-expression networks follow power-law distributions and are scale-free, small world networks. They are characterized by a small number of highly connected nodes. In order to find a conservatively small number of hub genes, we decided to use a cut-off value that is towards the right of the distribution. Threshold for the number of links for hub genes = Mean + 2 × Standard deviation = 40.7. Genes with a number of links ≥40 were considered hub genes. This approach resulted in 6.4% being hub genes in the entire network. [file gb-2008-9-10-r148-S6.pdf]
